# Association of Transit Time With Cold Ischemic Time in Kidney Transplantation

**DOI:** 10.1001/jamanetworkopen.2021.41108

**Published:** 2021-12-23

**Authors:** Andrew M. Placona, Casey Humphries, Chris Curran, Woodlhey Ambroise, Jeffrey P. Orlowski, Katrina Gauntt, Jen Wainright

**Affiliations:** 1 United Network for Organ Sharing, Richmond, Virginia; 2 LifeGift, Houston, Texas; 3 LifeShare Transplant Donor Services of Oklahoma, Inc, Oklahoma City

## Abstract

This cross-sectional study examines the association between transit time and cold ischemic time for adult, deceased kidney transplant donors.

## Introduction

The Organ Procurement and Transplant Network (OPTN) is developing an organ allocation framework called Continuous Distribution. This proposed allocation system would incorporate medical urgency, posttransplant survival, biological factors, access, and placement efficiency.^1^ It would derive a candidate’s score by weighting the scores of each subcomponent.

There are concerns about how to balance placement efficiency via cold ischemic time (CIT), defined as the time between cross-clamp and anastomosis, with other allocation goals.^2^ Placement efficiency is difficult to assess in kidney allocation because of the lack of organ transit data.^3,4^ Hence, understanding the association between organ transit time and CIT is important.

## Methods

In this cross-sectional study, we used data from the OPTN, which collects data on donors, wait-listed candidates, and transplant recipients in the US. The data reported were supplied by the United Network for Organ Sharing as the contractor for the OPTN. We identified 950 adult, deceased kidney transplant donors between August 2, 2019, and August 1, 2020, from whom kidneys were procured by LifeGift and LifeShare of Oklahoma.

We obtained institutional review board approval from Advarra, which declared the study exempt from the requirement for informed consent because the data were deidentified, in accordance with 45 CFR 46.104(d)(4). This study adheres to the Strengthening the Reporting of Observational Studies in Epidemiology (STROBE) reporting guideline for cross-sectional observational studies.

The Health Resources and Services Administration, US Department of Health and Human Services, provides oversight to the activities of the OPTN contractor. We received transportation details (ie, order details, pickup time, delivery time, and method of transportation) from the transportation vendor identified by both organ procurement organizations as their primary vendor. We performed a geospatial linkage, on the basis of the latitude and longitude of the vendor’s delivery address and the transplant center’s address, because the addresses did not exactly match. We ensured that our linkage was for the same donor within 24 hours of transplant date. We reviewed 339 linkages manually where kidney laterality was not available. We defined postprocurement time as the time between cross-clamp and vendor pickup from the donor hospital or recovery facility, transit time as between vendor pickup from the hospital or recovery facility and delivery to the transplant hospital, and transplant time as CIT minus postprocurement and transit time. After removing 18 observations with negative CIT and 5 with procurements occurring after vendor pickup time, we calculated Spearman rank correlations between transit time, postprocurement time, CIT, distance, and transplant time for 316 transplants, using 2-sided hypothesis tests with significance set at α < .05. We performed a subanalysis on 246 driven and 66 flown organs. We also examined a more restrictive geospatial link (1 mile) to test the robustness of our results. We performed our analyses in R statistical software, version 4.0.5 (R Project for Statistical Computing). Data were analyzed from October 2020 to September 2021.

## Results

Table 1 describes our data set, which included 316 adult, deceased kidney transplant donors. Although flown organs have higher CIT than driven organs, Table 2 illustrates that the correlation coefficient between transit time and CIT was low (*ρ* = 0.20; *P* < .001); in addition, our subanalysis of flown organs also demonstrated that the association between transit time and CIT was not significant (*ρ* = 0.16; *P* = .18). The correlation coefficient between distance and CIT (*ρ* = 0.21; *P* < .001) was higher than that between transit time and CIT. The correlation coefficient between distance and transit time was high (*ρ* = 0.67; *P* < .001). Both postprocurement time and transplant time were associated with CIT (postprocurement time: *ρ* = 0.63; *P* < .001; transplant time: *ρ* = 0.54; *P* < .001). Sensitivity analyses indicated our results were robust to our linkage definition.

**Table 1.** Descriptive Statistics of Study Data Set

Variable	Median (IQR)		
	Driven (n = 246)^a^	Flown (n = 66)^b^	Overall (N = 316)^c^
Cold ischemic time, h^d^	19.7 (15.5-24.0)	23.6 (19.5-28.4)	20.3 (16.3-25.1)
Distance, nautical miles^d^	204 (146-215)	814 (584-1060)	207 (173-382)
Procurement time, h^e^	7.4 (5.2-11.4)	7.3 (4.5-12.1)	7.4 (5.1-11.5)
Transit time, h^e^	4.6 (3.3-5.4)	8.3 (6.6-10.2)	4.9 (3.6-6.4)
Transplant time, h^e^	6.3 (4.0-9.5)	5.1 (3.2-10.4)	6.0 (3.7-9.5)
Kidney donor profile index^e^	46.5 (24.0-0.74.5)	32.5 (19.0-50.5)	43.0 (22.0-70.0)
Biopsy performed, No. (%)			
No	87 (35)	33 (50)	121 (38)
Yes	159 (65)	33 (50)	195 (62)
Kidney pumped, No. (%)			
No	47 (19)	31 (47)	80 (25)
Yes	199 (81)	35 (53)	236 (75)

^a^ Based on organs that vendor identified as driven.

^b^ Based on organs that vendor identified as flown.

^c^ Based on all observations. Some organs had other travel modalities (n = 4).

^d^ Derived from Organ Procurement and Transplant Network data.

^e^ Derived from transportation vendor data. The kidney donor profile index combines a variety of donor factors into a single number that summarizes the likelihood of graft failure after deceased donor kidney transplant. The value ranges from 0 to 100.

**Table 2.** Correlation Matrix of Components of Time (Driven, Flown, Overall)

Travel modality	ρ				
	Postprocurement	Transit	Transplant	Distance	CIT
Driven (n = 246)^a^					
Postprocurement^b^	NA	NA	NA	NA	NA
Transit^b^	−.15^c^	NA	NA	NA	NA
Transplant^b^^,^^d^	.05	−.00	NA	NA	NA
Distance^d^	−.21^e^	.61^e^	.10	NA	NA
CIT^d^	.68^e^	.14^c^	.64^e^	.07	NA
Flown (n = 66)^f^					
Postprocurement^b^	NA	NA	NA	NA	NA
Transit^b^	−.13	NA	NA	NA	NA
Transplant^b^^,^^d^	−.26^c^	−.03	NA	NA	NA
Distance^d^	.06	.07	.35^g^	NA	NA
CIT^d^	.63^e^	.16	−.11	−.05	NA
Overall (N = 316)^h^					
Postprocurement^b^	NA	NA	NA	NA	NA
Transit^b^	−.17^g^	NA	NA	NA	NA
Transplant^b^^,^^d^	−.02	−.07	NA	NA	NA
Distance^d^	−.13^c^	.67^e^	−.00	NA	NA
CIT^d^	.63^e^	.20^e^	.54^e^	.21^e^	NA

Abbreviations: CIT, cold ischemic time; NA, not applicable.

^a^ Based on organs that vendor identified as driven.

^b^ Derived from transportation vendor data.

^c^ *P* ≤ .05 (2-sided).

^d^ Derived from Organ Procurement and Transplant Network data.

^e^ *P* ≤ .001 (2-sided).

^f^ Based on organs that vendor identified as flown.

^g^ *P* ≤ .01 (2-sided).

^h^ Based on all observations. Some organs had other travel modalities (n = 4).

## Discussion

To our knowledge, this cross-sectional study was the first to quantify the subcomponents of CIT and aligned with previous research on CIT and distance.^5^ Our data suggested that transit time had a low correlation with CIT, but transplant and postprocurement time were moderately correlated with CIT.

A limitation of our study was that we obtained transit data on adult kidney transplants from 2 organ procurement organizations. The association between transit time and CIT may have varied by both geographical region and the organ in question. These findings are important given the impact of transit and CIT on organ allocation.^6^ Efforts to ensure low CIT should focus on identifying and reducing practices associated with increased postprocurement and transplant time.
